# Regulatory Cell Populations in Relapsing-Remitting Multiple Sclerosis (RRMS) Patients: Effect of Disease Activity and Treatment Regimens

**DOI:** 10.3390/ijms17091398

**Published:** 2016-08-25

**Authors:** Maria Rodi, Nikolaos Dimisianos, Anne-Lise de Lastic, Panagiota Sakellaraki, George Deraos, John Matsoukas, Panagiotis Papathanasopoulos, Athanasia Mouzaki

**Affiliations:** 1 Division of Hematology, Department of Internal Medicine, Faculty of Medicine, University of Patras, Patras GR-26500, Greece; marodi_biol@yahoo.gr (M.R.); delastic@gmail.com (A.-L.d.L.); gsakel@upatras.gr (P.S.); 2 Department of Neurology, Faculty of Medicine & University Hospital, University of Patras, Patras GR-26500, Greece; ndimisianos@yahoo.gr (N.D.); papat@upatras.gr (P.P.); 3 Eldrug S.A., Pharmaceutical Company, Platani, Patras GR-26504, Greece; gderaos@upatras.gr (G.D.); imats@upatras.gr (J.M.)

**Keywords:** multiple sclerosis, Tregs, HLA-G, iNKT, NK^bright^, methylprednisolone, natalizumab, interferon, myelin oligodendrocyte glycoprotein, MOG, myelin basic protein, MBP

## Abstract

Multiple sclerosis (MS) is a demyelinating disease of the central nervous system (CNS) of autoimmune etiology that results from an imbalance between CNS-specific T effector cells and peripheral suppressive mechanisms mediated by regulatory cells (RC). In this research, we collected blood samples from 83 relapsing remitting MS (RRMS) patients and 45 healthy persons (HC), to assess the sizes of their RC populations, including CD4^+^CD25^high^Foxp3^+^ (nTregs), CD3^+^CD4^+^HLA^−^G^+^, CD3^+^CD8^+^CD28^−^, CD3^+^CD56^+^, and CD56^bright^ cells, and how RC are affected by disease activity (acute phase or remission) and types of treatment (methylprednisolone, interferon, or natalizumab). In addition, we isolated peripheral blood mononuclear cells (PBMC) and cultured them with peptides mapping to myelin antigens, to determine RC responsiveness to autoantigens. The results showed decreased levels of nTregs in patients in the acute phase ± methylprednisolone and in remission + natalizumab, but HC levels in patients in remission or receiving interferon. Patients + interferon had the highest levels of CD3^+^CD4^+^HLA^−^G^+^ and CD3^+^CD8^+^CD28^−^ RC, and patients in the acute phase + methylprednisolone the lowest. Patients in remission had the highest levels of CD3^+^CD56^+^, and patients in remission + natalizumab the highest levels of CD56^bright^ cells. Only nTregs responded to autoantigens in culture, regardless of disease activity or treatment. The highest suppressive activity was exhibited by nTregs from patients in remission. In conclusion, in RRMS disease activity and type of treatment affect different RC populations. nTregs respond to myelin antigens, indicating that it is possible to restore immunological tolerance through nTreg induction.

## 1. Introduction

Multiple sclerosis (MS) is a chronic autoimmune/inflammatory disease of the central nervous system (CNS) that results in the demyelination of neurons leading to axonal loss and the accumulation of disability [1]. MS usually affects young adults aged between 20 and 40 years; women are affected at least twice as often as men [2]. The course of MS can follow four clinical patterns that include relapsing remitting MS (RRMS, which accounts for 80%–90% of MS cases at onset), secondary progressive MS (SPMS), primary progressive MS (PPMS) and progressive relapsing MS (PRMS) [3,4]. MS is characterized by effector T-cell (Teff) and macrophage infiltrates that are triggered by CNS-specific CD4^+^ T-cells, and autoantibody production [5]. The main autoimmune etiology of MS consists of activated IFN-γ-producing T helper 1 (Th1) cells, that recognize peptides of the myelin sheath, including myelin basic protein (MBP), proteolipid protein (PLP), and myelin oligodendrocyte glycoprotein (MOG) [3], as well as IL-17-producing Th17 cells, a subset of CD4^+^ T-cells shown to be involved in the pathogenesis of autoimmune diseases [6]. B-cells also play a significant role in the pathogenesis of MS not only because they produce autoantibodies, but because they can stimulate autoreactive CD4^+^ T-cells directly, through self-antigen presentation and the production of proinflammatory cytokines [7]. Current theories concerning the pathogenesis of MS involve genetic and environmental factors, as well as immune dysregulation. Autoreactive T-cells and antibodies are found in both patients with autoimmune diseases and healthy individuals, so their mere presence is not enough for the development of an autoimmune response. The breakdown of immune tolerance to CNS self-antigens in genetically susceptible individuals is considered a key event in the development of MS [4,5,8].

Experimental reports have demonstrated the important role of regulatory T-cells (Tregs) in CNS autoimmunity [8,9,10]. The types of Tregs shown to exert regulatory activities in the CNS include natural Tregs (nTregs), which are CD4^+^ T-cells that arise in the thymus and constitutively express the CD25 cell marker (CD25^high^) and the transcription factor forkhead box protein P3 (Foxp3) and inducible Tregs (iTregs), which can be induced in the periphery during an autoimmune or inflammatory response, and may or may not express Foxp3 [8,9,10]. iTregs include T helper 3 (Th3) cells, which originate from naive T-cells that are either CD4^+^ or CD8^+^ and secrete TGF-β, type 1 Tregs (Tr1) cells, which are derived from CD4^+^ precursors and secrete IL-10, and CD8^+^CD28^−^ cells that render antigen presenting cells (APC), mainly dendtritic cells (DCs), tolerant through cell-cell contact, can secrete IL-10, TGF-β, IFN-γ, CCL4, and directly kill CD4^+^ Teffs and APCs [8,9,10,11]. Although Foxp3 is considered a specific marker of nTregs, activated Teffs and Tr1 cells can transiently express Foxp3 [12,13].

Other, less studied, populations of regulatory cells include CD4^+^ or CD8^+^ T-cells that lack Foxp3 expression but express the human leukocyte antigen G (HLA-G) [8,14], natural killer T regulatory cells (iNKT) and CD56^bright^ NK cells, that suppress autoreactive Teffs through direct cytotoxicity and/or cytokine secretion [15,16].

Several studies have addressed the issues of numbers and function of Tregs in MS patients, focusing mainly on CD4^+^CD25^high^ T-cells. These studies have found no differences in the frequencies of Tregs between MS patients and controls or between patients at different disease stages (acute phase or remission) or with different disease subtypes (clinically isolated syndrome (CIS), RRMS, SPMS, PPMS), although they found loss of Treg suppressive activity [17,18,19]. Studies investigating CD4^+^CD25^high^Foxp3^+^ Tregs in MS patients showed functional impairment [20,21,22] linked to reduced Foxp3 expression [20,22] and reduced frequency of Foxp3^+^ cells [22], whereas other investigators found decreased levels during remission, which were restored to normal levels during the acute phase of the disease [23].

Interferon β (IFN-β) has long been established as the first line immunomodulatory treatment for patients with RRMS. Although the exact mechanism of action for preventing relapses remains elusive, several studies have suggested that this could be mediated by restoring the levels and function of Tregs [24,25,26,27,28]. MS relapses are treated with high-dose, short-term intravenous glucocorticoids (e.g., methylprednisolone). It has been suggested that glucocorticoids mitigate relapses by restoring the suppressive function of Tregs [29,30].

Natalizumab is a monoclonal antibody against the α4 subunit of α4β1 (very late antigen-4, VLA-4) and α4β7 integrins, located on the surface of lymphocytes, that acts by blocking their binding to their endothelial receptors, vascular cell adhesion molecule-1 (VCAM-1) and mucosal addressin-cell adhesion molecule-1 (MadCAM-1), respectively. This blocking inhibits the infiltration of autoreactive T-cells into the CNS through the blood-brain barrier (BBB) and, thus, suppresses CNS tissue inflammation [31]. Natalizumab has been approved for the prophylactic treatment of RRMS patients who have failed to respond to first-line therapies or have severe, breakthrough disease [32,33]. Although natalizumab has been shown to affect immune cell responses in more ways than just preventing infiltration of T-cells into the CNS, very little is known regarding its effects on Tregs.

In this work we assessed the frequencies of peripheral blood CD4^+^CD25^+^Foxp3^+^ (nTregs), CD3^+^CD4^+^HLA^−^G^+^, CD3^+^CD56^+^ (iNKT cells), CD8^+^CD28^−^ (CD8^+^ Tregs) and CD56^bright^ cells in RRMS patients and healthy controls, to determine the effects of disease activity (acute or stable disease) and treatment regimens (methylprednisolone, interferon, natalizumab) thereof. In addition, we tested regulatory cell responses to various antigenic peptides mapping to myelin epitopes, to assess their function in patients at different states of the disease and under different therapies.

## 2. Results

### 2.1. CD4^+^CD25^high^Foxp3^+^ T-Cells (nTregs) in RRMS

**Figure 1:**
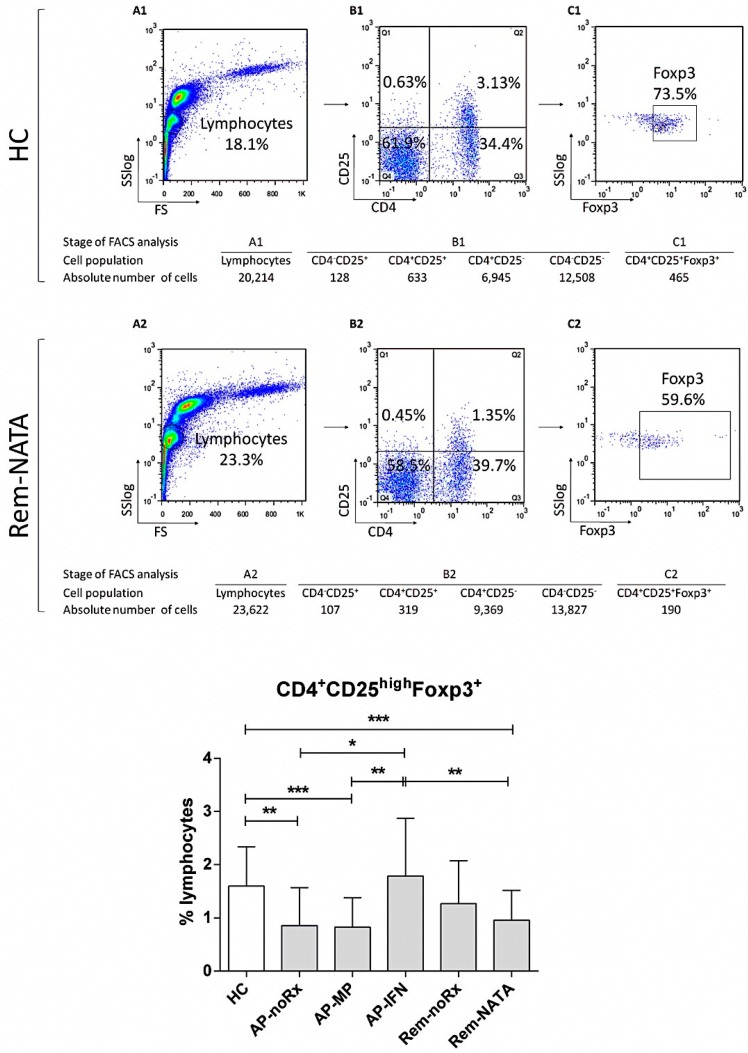
Flow cytometric analysis to determine CD4^+^CD25^+^Foxp3^+^ RC levels in human peripheral blood. A representative analysis is shown for one healthy control (**A1**–**C1**) and one relapsing remitting MS (RRMS) patient (**A2**–**C2**). The white blood cells (WBC) were gated on lymphocytes, based on forward and side light scatter (**A1**,**A2**) and analyzed for CD4 and CD25 expression (**B1**,**B2**); The double positive cells were analyzed further for Foxp3 expression (**C1**,**C2**); The numbers in the dot plots indicate the percentage of gated cells expressing the relevant marker. The tables underneath, show the absolute number of cells in each population analyzed; **Bottom graph**: The results of the analysis of all patients (*n* = 83) and controls (HC, *n* = 45). AP-noRx (*n* = 13), patients in the acute phase of the disease without treatment; AP-MP (*n* = 17), patients in the acute phase under treatment with methylprednisolone; AP-IFN (*n* = 12), patients in the acute phase under treatment with interferon β; Rem-noRx (*n* = 15), patients in remission receiving no treatment; Rem-NATA (*n* = 26), patients in remission under treatment with natalizumab. * *p* = 0.04, ** *p* = 0.01, *** *p* = 0.002.

The levels of nTregs in peripheral blood of MS patients and HC, are shown in Figure 1. nTreg levels were lower in acute-phase patients with no treatment (AP-noRx) or under methylprednisolone (AP-MP), compared to all other patient groups and HC. nTregs were restored to normal levels in acute-phase patients under interferon β treatment (AP-IFN), whereas patients in remission under no treatment (Rem-noRx) showed intermediate levels between HC and AP-noRx. Patients in remission under natalizumab (Rem-NATA) had nTreg levels comparable to those of AP-noRx and AP-MP patients (Figure 1D).

### 2.2. Other Regulatory Cell Populations in RRMS

**Figure 2:**
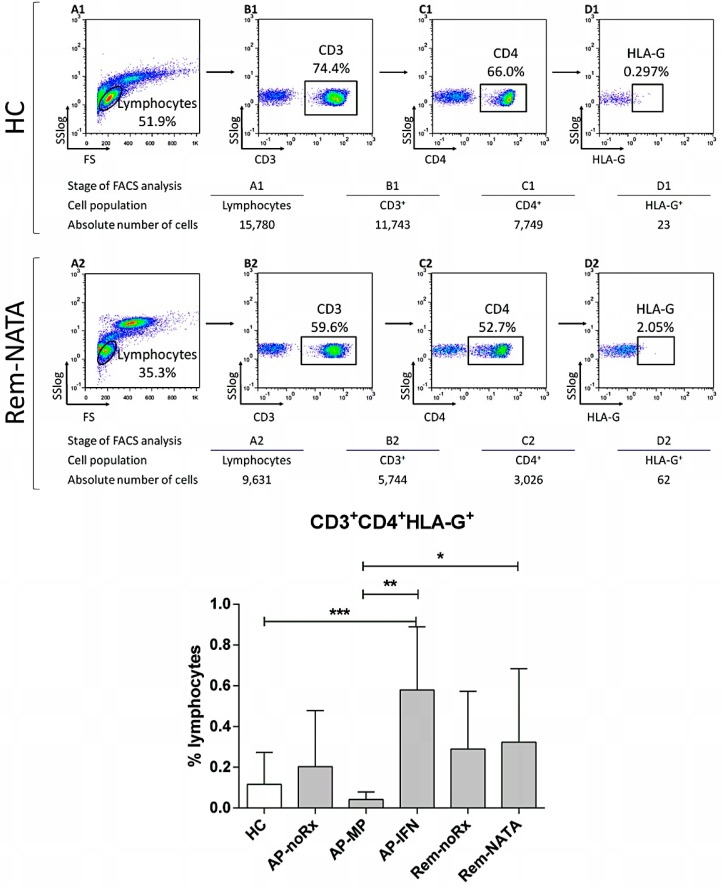
Flow cytometric analysis to determine CD3^+^CD4^+^HLA^−^G^+^ RC levels in human peripheral blood. A representative analysis is shown for one healthy control (**A1**–**D1**) and one RRMS patient (**A2**–**D2**); The WBC were gated on lymphocytes, based on forward and side light scatter (**A1**,**A2**) and analyzed for CD3 (**B1**,**B2**), CD4 (**C1**,**C2**) and HLA-G expression (**D1**,**D2**); The numbers in the dot plots indicate the percentage of gated cells expressing the relevant marker. The tables underneath show the absolute number of cells in each population analyzed; **Bottom graph:** The results of the analysis of all patients (*n* = 83) and controls (HC, *n* = 45). For abbreviations see legend of Figure 1. * *p* = 0.05, ** *p* = 0.01, *** *p* = 0.006.

**Figure 3:**
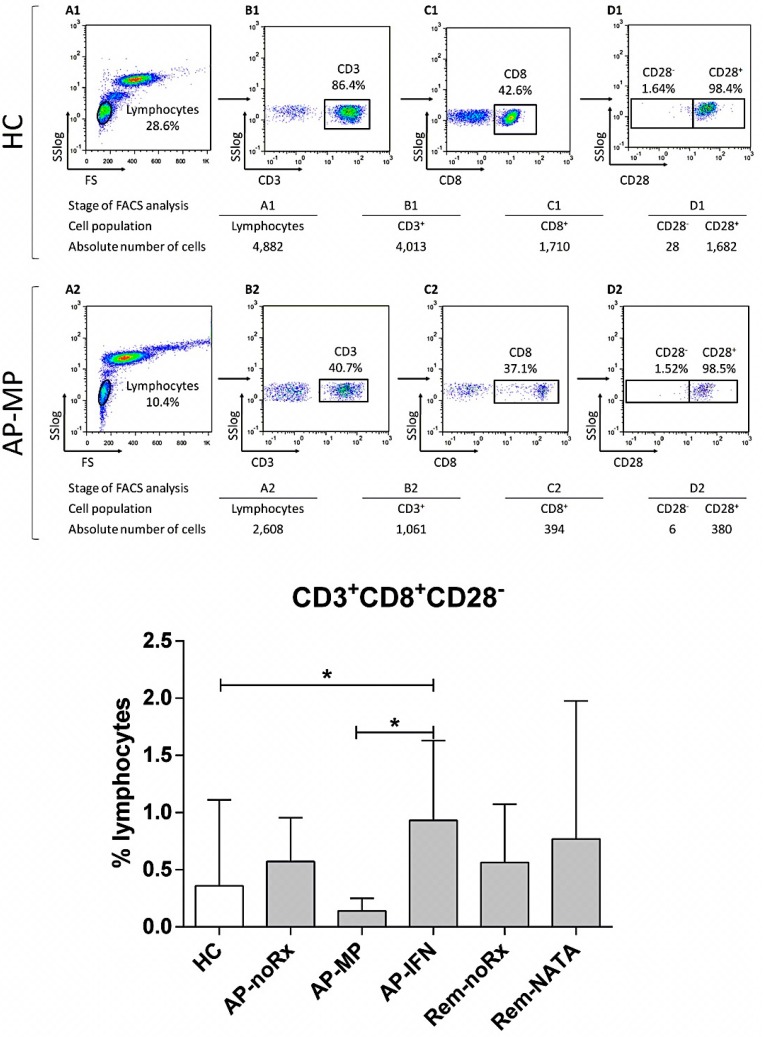
Flow cytometric analysis to determine CD3^+^CD8^+^CD28^−^ RC levels in human peripheral blood. A representative analysis is shown for one healthy control (**A1**–**D1**) and one RRMS patient (**A2**–**D2**); The WBC were gated on lymphocytes, based on forward and side light scatter (**A1**,**A2**) and analyzed for CD3 (**B1**,**B2**), CD4 (**C1**,**C2**), and CD28 (**D1**,**D2**) expression. The numbers in the dot plots indicate the percentage of gated cells expressing the relevant marker. The tables underneath, show the absolute number of cells in each population analyzed; **Bottom graph**: The results of the analysis of all patients (*n* = 83) and controls (HC, *n* = 45). For abbreviations see legend of Figure 1. * *p* = 0.05.

**Figure 4:**
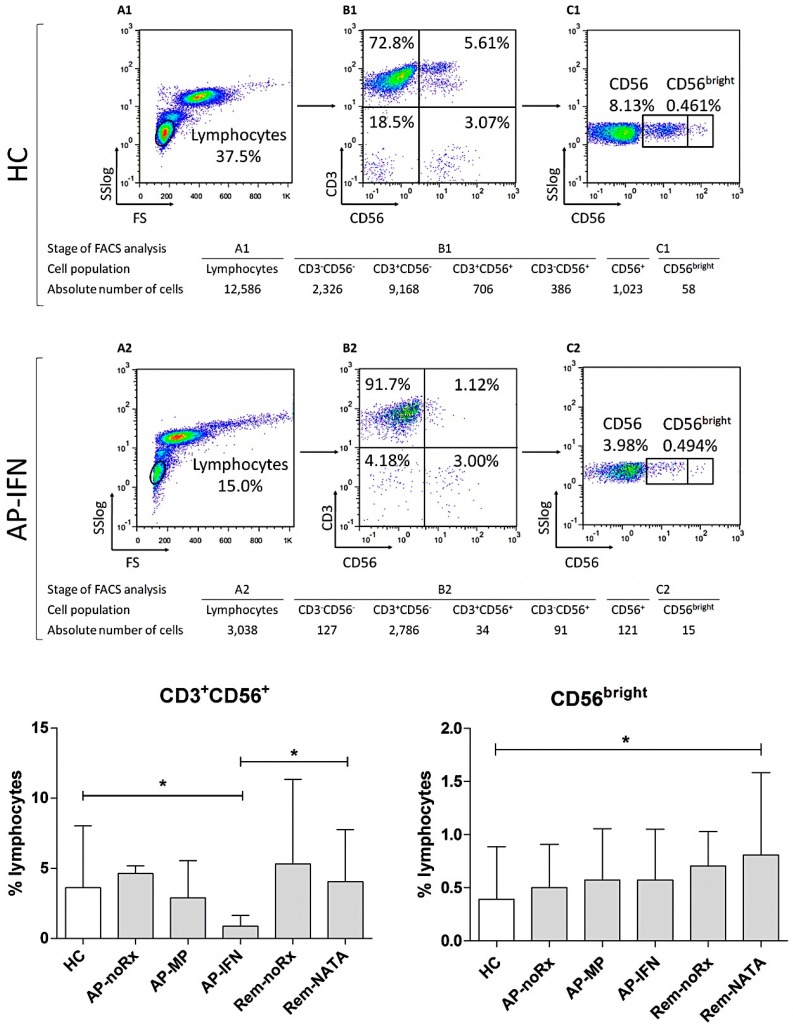
Flow cytometric analysis to determine CD3^+^CD56^+^ and CD56^bright^ RC levels in human peripheral blood. A representative analysis is shown for one healthy control (**A1**–**C1**) and one RRMS patient in the acute phase under interferon β (AP-IFN) treatment (**A2**–**C2**); The WBC were gated on lymphocytes, based on forward and side light scatter (**A1**,**A2**), and analyzed for CD3 and CD56 expression (**B1**,**B2**); The CD56 positive cells were further analyzed for the intensity of CD56 expression (CD56^bright^) (**C1**,**C2**); The numbers in the dot plots indicate the percentage of gated cells expressing the relevant marker. The tables underneath, show the absolute number of cells in each population analyzed; **Bottom graphs**: The results of the analysis of all patients (*n* = 83) and controls (HC, *n* = 45). (**Left**) CD3^+^CD56^+^; (**Right**) CD56^bright^. For abbreviations see legend of Figure 1. * *p* = 0.05.

The levels of the other regulatory cell populations studied in the peripheral blood of MS patients and HC, are shown in Figure 2, Figure 3 and Figure 4. The percentage of CD3^+^CD4^+^HLA^−^G^+^ T-cells was higher in AP-IFN patients compared to all other groups, with the difference reaching statistical significance between AP-IFN and HC, AP-MP, and Rem-NATA patients (Figure 2).

The CD3^+^CD8^+^CD28^−^ T-cells (CD8^+^ Tregs) showed the same pattern, with the AP-IFN patients having the highest levels of CD8^+^ Tregs in all groups (Figure 3). The opposite occurred with CD3^+^CD56^+^ cells (iNKT cells), with the AP-IFN patients showing the lowest frequency (Figure 4). The frequency of CD56^bright^ cells was higher in all patient groups compared to HC, but only the difference between Rem-NATA patients and HC was statistically significant (Figure 4).

### 2.3. Effect of Culture with Peptides on Regulatory Cell Function

Peripheral blood mononuclear cells (PBMC) were isolated from RRMS patients (AP-noRx, *n* = 5; AP-MP, *n* = 5; AP-IFN, *n* = 6; Rem-noRx; *n* = 5, Rem-NATA, *n* = 14) and controls (HC, *n* = 19) and cultured in the presence or absence of peptides mapping to myelin antigens (see M and M), to assess which RC populations responded to autoantigens. It was observed that only nTregs responded to the peptides by proliferation and cytokine secretion, whereas the other RC populations studied did not respond to the peptides (data not shown).

**Figure 5:**
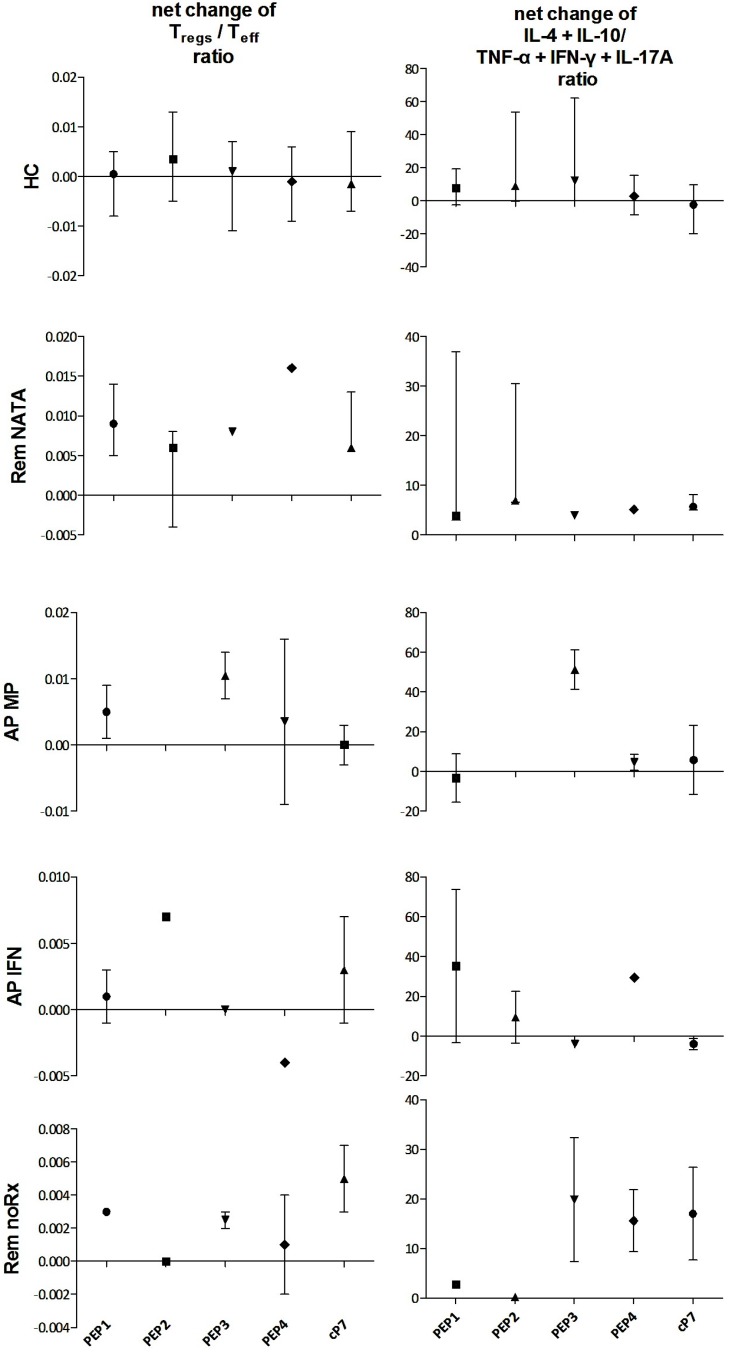
**Left column:** Net % changes of regulatory/effector ratios (CD4^+^CD25^+^/CD4^+^CD25^−^) after 72 h culture of PBMC of MS patients and HC with the antigenic peptides PEP1-4 or cP7; **Right column**: Net % changes in the corresponding anti-inflammatory/inflammatory cytokine ratios ([IL-4 + IL-10]/[IFN-γ + TNF-α + IL-17A]) in the culture supernatants.

To delineate the effects of disease state and treatment regimens on nTreg responses, the numbers of CD4^+^CD25^−^ and CD4^+^CD25^+^ cells were measured in cultures ± peptides, to calculate the ratio of effector to suppressor cells, as well as the percentage of CD4^+^CD25^+^Foxp3^+^ nTregs. The net change of effector/suppressor ratios between cultures ± peptides was also calculated, to assess any shift towards an effector or suppressor phenotype. Positive changes reflected an effector shift whereas negative a suppressor shift (Figure 5, **left column**). These changes were accompanied by corresponding changes in cytokine ratios, with an increase in anti-inflammatory cytokines when a suppressor shift was observed (Figure 5, **right column**).

**Figure 6:**
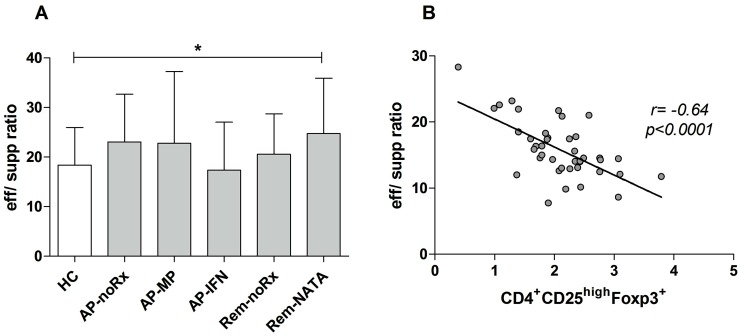
(**A**) Effector/suppressor ratios of HC and patient groups after cultures of PBMC in the absence of antigenic peptides. Note the complementarity with the CD4^+^CD25^high^Foxp3^+^ frequencies in Figure 1. * *p* = 0.05; and (**B**) correlation of effector/suppressor ratios in culture, with the percentage of corresponding CD4^+^CD25^high^Foxp3^+^ Tregs (Spearman *r* = −0.64, *p* < 0.0001).

Effector/suppressor ratios in cultures-peptides showed a reverse trend to that of nTregs in peripheral blood (Figure 6). The higher ratios were observed in AP-noRx, AP-MP, and Rem-NATA patients, and the lower in AP-IFN patients (Figure 6A). This observation was confirmed by a strong inverse correlation between effector/suppressor ratios and CD4^+^CD25^+^Foxp3^+^ T-cells (*r* = −0.64, *p* < 0.0001) (Figure 6B).

**Figure 7:**
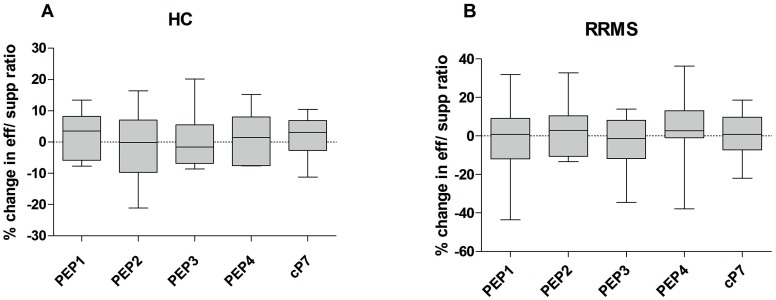
Mean changes in effector/suppressor ratios in HC (**A**) and RRMS patients (**B**), after culture with the antigenic peptides (PEP1-4, cP7) (whiskers are min to max).

Overall, all groups of RRMS patients displayed a suppressive phenotype during co-cultures with one or more antigenic peptides, similarly to the HC group. Peptides PEP2 and PEP3 were relatively more tolerogenic for HC subjects and PEP1 and PEP3 for RRMS patients (Figure 7).

**Figure 8:**
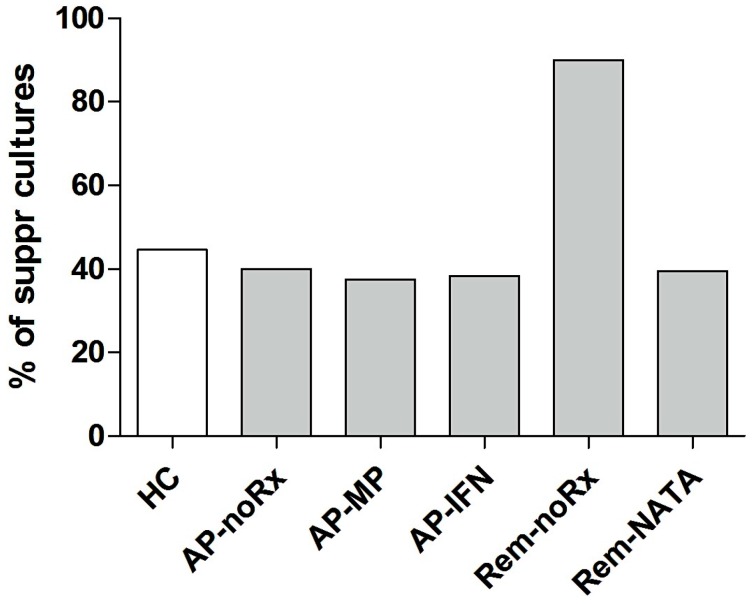
Percentage of cultures showing a suppressive shift in HC and patient groups, after culture with the antigenic peptides (PEP1-4, cP7). Note the significant increase in Rem-noRx patients.

When the percentages of co-cultures with a suppressive change in effector/suppressor ratio were calculated, no statistical differences between patient groups and HC were found, with the exception of the Rem-noRx patient group that showed a more than two-fold higher percentage of suppressive cultures compared to the other patient groups and HC (Figure 8).

## 3. Discussion

The role of Tregs in the pathogenesis of MS and other autoimmune diseases, is investigated by many groups, mainly because of their putative therapeutic potential [8,9,10]. In earlier years, the only marker used to differentiate Tregs from effector CD4^+^ T-cells was the constitutive expression of CD25 (IL-2Rα chain) [34], although it was already recognized that the pool of CD4^+^CD25^+^ Tregs could include recently activated Teffs. However, CD4^+^ T-cells that express high levels of CD25 (CD25^high^) exhibit the highest suppressive activity and represent a small percentage (2%–4%) of CD4^+^ T-cells [34]. Earlier studies of CD4^+^CD25^high^ Tregs in MS, showed similar frequencies of these cells in the peripheral blood between MS patients and healthy controls [17,18,19,28], but reported reduced suppressive activity using various functional assays [18,19,28]. In the CSF, compared to blood, CD4^+^CD25^high^ Tregs were reported to be similar [19], or elevated [28] in MS patients. Subsequent studies used additional markers to characterize nTregs from the CD4^+^CD25^high^ pool, the most important of which was the expression of Foxp3. Foxp3 is considered a specific marker for nTregs, although Teffs and Tr1 cells can transiently express Foxp3 upon activation [13], through STAT-5 signaling cytokines [12].

Other markers used for the characterization of Tregs include CD39 (an ectoenzyme that degrades ATP to AMP), CTLA-4 (Cytotoxic T-lymphocyte antigen 4, a CD28-family receptor) and GITR (Glucocorticoid-induced tumor necrosis factor receptor, a member of the TNF receptor superfamily) [23,35]. Huan et al. [20] reported identical levels of CD4^+^CD25^+^ Tregs in RRMS patients and HC, but reduced expression of Foxp3 in RRMS patients. Reduced levels of CD4^+^CD25^high^Foxp3^+^ Tregs in the blood of RRMS (but not SPMS) patients, with an associated increase of Tregs in the CSF, were also observed by Venken et al. [22]. Michel et al. [36] reported that the CD4^+^CD25^high^ pool of T-cells was depleted of cells expressing IL-7 receptor α-chain (CD127), a marker present on activated T-cells but not on Tregs. CD4^+^CD25^high^CD127^low^ Tregs exhibited similar suppressive functions in RRMS patients and HC. Dalla Libera et al. [23] reported decreased numbers of Tregs (defined by CD25, Foxp3, CD39, CTLA-4, and GITR expression) in RRMS patients during remission, which were restored to normal levels in the acute phase, concluding that Tregs are not involved in causing clinical attacks, but retain functionality and are increased during acute phase to restore homeostasis. Bjerg et al. [37] studied RRMS patients in remission and found that CD4^+^CD25^high^CD127^low^Foxp3^+^ Tregs were higher than HC in the group of patients with less severe disease (lower EDSS score and shorter disease duration) and lower in patients with more severe disease.

Other studies addressed the issue of the effect of immunomodulating and immunosuppressive treatments on Tregs. One study with IFNβ-1α-treated MS patients, showed increased numbers of CD4^+^CD25^high^ Tregs (CTLA-4^+^ and GITR^+^) and functional enhancement after six months of treatment [38]. Other studies with RRMS patients treated with IFNβ (β-1α or β-1β), found improved frequency and function of CD4^+^CD25^+^Foxp3^+^ Tregs [27,28]. Natalizumab treatment was reported to have no effect on CD4^+^CD25^high^Foxp3^+^ Tregs frequency or activity [21,39], with the exception of one case study that reported increased numbers and restored function of nTregs in a patient treated with natalizumab after relapsing following stem cell transplantation [40]. Treatment with glucocorticoids after an acute attack was reported to induce an increase in CD4^+^CD25^high^ [29] and Foxp3^+^ Treg frequency and function [30].

The marked diversity in the observations between various studies regarding population sizes, disease characteristics, comparators, Treg identification markers, and methodology used for Treg isolation and functional assays, make a direct comparison of the results very difficult. Our results showed decreased levels of CD4^+^CD25^high^Foxp3^+^ Tregs in patients in the acute phase (except when under IFNβ treatment) and an up-regulation during remission (except when under natalizumab). This is in contrast to the observations by Dalla Libera et al. [23] who reported lower levels during stable disease and restoration to control levels during acute clinical attacks. However, the authors did not clarify how many of their patients were under prophylactic treatment, if any, and they also used mRNA expression of Foxp3 and other Treg markers within the CD4^+^ T-cells, which are also expressed by activated Teffs and Tr1 cells [13]. Haas et al. [19] found decreased suppressive activity of CD4^+^CD25^high^ Tregs in their RRMS patients in the acute phase. In a minor subset of patients entering remission, suppressive function of Tregs displayed an increase, though not statistically significant. The majority of the studies included patients in remission, so there was no acute phase arm to compare [17,18,20,22,37]. Reduced frequency of nTregs during the acute phase can be explained by their migration to sites of inflammation in the CNS, as previously suggested [22,41,42].

Our finding of increased frequency of nTregs under IFNβ treatment is consistent, since most studies verify it [22,24,26,27,28,38,43]. The fact that our patients were in the acute phase of the disease indicates that IFNβ treatment per se increases nTreg numbers regardless of disease activity. In addition, the effector/suppressor cell ratio of the patients under IFNβ treatment was similar to the HC group (cf. Figure 6) and their nTregs retained their function (cf. Figure 8). Nevertheless, these events were not by themselves sufficient to protect these patients from relapsing, perhaps because higher numbers of nTregs and/or nTregs with higher suppressive potential are needed, compared to HC, to ameliorate the disease by counteracting the activity of the autoreactive clones.

We also found that treatment with natalizumab does not restore the frequency of nTregs, a finding corroborated by others [21,39]. Patients in remission under natalizumab had also a significantly higher effector/suppressor cell ratio compared to HC (cf. Figure 6), probably reflecting the inhibition of Teff infiltration into the CNS through the BBB, although their nTregs retained their function (cf. Figure 8). It appears that in this group of patients peripheral blood nTreg levels, per se, are not reflective of disease activity and remission is due to natalizumab activity [31] and, perhaps, the effect of other RC populations that were elevated (see below).

Regarding the effect of glucocorticoids (methylprednisolone), we did not find any increase in nTregs compared to untreated acute phase patients, as indicated by others [29,30]. The reduced frequency of nTregs in patients in the acute phase under methylprednisolone treatment is, perhaps, due to nTreg migration to sites of inflammation in the CNS [22,41,42] that is reversed when the patients enter remission. Again, in these patients nTregs retained their function (cf. Figure 8).

In our study we also examined the frequencies of other RC phenotypes, which may play a role in regulating autoimmune diseases. HLA^−^G^+^ and CD8^+^ Tregs were elevated mainly in the AP-IFN group, whereas iNKT cells were markedly down-regulated in the same group. HLA^−^G^+^ Tregs have been recently identified as a novel subset of naturally, thymus-derived regulatory T-cells, found in the blood and sites of inflammation to modulate inflammatory responses [14,44]. CD8^+^ Tregs are also important in immune regulation and treatment with glatiramer acetate, another first-line therapy for RRMS, has been shown to enhance their function [45]. Natalizumab-treated patients in our study, while having lower levels of Foxp3^+^ Tregs, showed higher levels of HLA^−^G^+^ and CD8^+^ Tregs, as well as CD56^bright^ NK-cells, a subset of NK-cells with suppressive activity [46], the expansion of which is proposed as a mechanism of action of a new therapy for RRMS with a monoclonal antibody to CD25 (daclizumab) [47].

Our results from the functional assays showed that only nTregs, isolated from RRMS patients and HC, exhibited suppressive activity when cultured with various MOG or MBP peptides. This does not mean that the other RC populations studied in this work are not functional, merely that they do not respond to this type of antigenic stimulation. Under these culture conditions, nTregs from RRMS patients exhibited suppressive activity equal to nTregs isolated from HC or, as in the case of nTregs from patients in remission without therapy, higher than in HC. These results indicate that the reduction in nTreg suppressive activity reported in earlier studies [18,19] can be attributed to the type of stimulus used for the functional assays, and that it is possible to restore immunological tolerance through nTreg induction when suitable antigenic stimuli are employed.

## 4. Materials and Methods

### 4.1. Study Subjects

Eighty three patients with clinically definite MS of the relapsing-remitting type (RRMS), according to the 2005 revised McDonald’s criteria [48], were included in the study. Historical data and neurologic examination were used to assess their disease course, current status, disease duration, and level of disability, according to the expanded disability status scale-EDSS [49]. The demographic and clinical characteristics of MS patients are presented in Table 1. The patients were divided into five groups based on the disease status (acute phase, AP, or remission, Rem) and the type of treatment they were receiving at the time their blood samples were drawn (no treatment, noRx; methylprednisolone, MP; interferon β, IFN or natalizumab, NATA). The groups were: (a) patients in the acute phase of the disease who were treatment-naive (AP-noRx, *n* = 13); (b) patients in the acute phase under treatment with methylprednisolone (AP-MP, *n* = 17); (c) patients in the acute phase under treatment with interferon β (AP-IFN, *n* = 12); (d) patients in remission receiving no treatment (Rem-noRx, *n* = 15); and (e) patients in remission under treatment with natalizumab (Rem-NATA, *n* = 26). To note, in patients with clinically definite RRMS the acute phase is defined as a clinical relapse, i.e., a new onset of a neurological dysfunction of the kind seen in MS that can be either a subjective report (symptom) or an objective observation (sign). Usually it is both, since a new symptom indicates a dysfunction that can be identified by the neurologist. The duration of the new symptom or sign has to be longer than 24 h, and usually lasts for days or weeks. It is not necessary to perform an MRI to verify the relapse; it is, per se, a clinical event.

Forty five age- and sex-matched, healthy individuals (Table 1), with no history of neurological or autoimmune disease and no concomitant signs or symptoms of infection or inflammation, served as a healthy control group (HC). All subjects signed an informed consent form before blood collection. The study protocol was approved by the Scientific Review Board and Ethics Committee of Patras University Hospital (Reg# 451/17.10.08, 17 October 2008). The Hospital abides by the Helsinki declaration on ethical principles for medical research involving human subjects.

### 4.2. Cells and Cultures

Whole blood samples (10 mL) were collected from MS patients and HC in heparinized BD Vacutainers (BD, Plymouth, UK). Peripheral blood mononuclear cells (PBMC) were isolated by ficoll (BIOCHROM AG, Berlin, Germany) density gradient centrifugation. The cells were cultured for 72 h in RPMI 1640 medium (GIBCO BRL, Gaithersburg, MD, USA), containing 10% fetal bovine serum and 1% penicillin/streptomycin at a concentration of 10^6^ cells/mL, in the presence or absence of peptides PEP1-4 or cP7 (see Section 4.3), at a concentration of 10 pg/mL/10^6^ cells. At the end of the culture period, PBMC were harvested and the culture supernatants were collected for the determination of cytokine concentrations (see Section 4.5). PBMC were washed with PBS and their numbers and phenotypes were determined by flow cytometry.

### 4.3. Peptides Mapping to MOG and MBP Myelin Antigens

The peptides used for the functional assays are shown in Table 2. Peptides PEP1-4 are MOG_35–55_ antigenic epitopes prepared as described [50]. Peptide cP7 is an MBP_87–99_ antigenic epitope prepared as described [51].

### 4.4. Flow Cytometry

Whole blood cells or PBMC were labeled using the following monoclonal antibodies: CD3-PC5 (UCHT1, Beckman Coulter-BC, Paris, France), CD4-FITC (13B8.2, BC), CD8-FITC (B9.11, BC), CD28-PE (B-T3, Abcam, Cambridge, UK), CD25-PE (M-A251, Becton Dickinson-BD Biosciences-Pharmigen, San Diego, CA, USA), CD56-PE (N901, BC), HLA-G-PE (MEM-G/9, Abcam), and PE-Cy5 Conjugated anti-human Foxp3 (PCH101, eBiosciences, San Diego, CA, USA). All procedures were performed according to the manufacturers’ instructions. For intracellular staining of Foxp3, the cells were labeled with CD4-FITC and CD25-PE antibodies and were stained intracellularly for Foxp3, after fixation and permeabilization. Whole blood samples were treated with BD Pharm Lyse buffer 1× (BD) for 15 min, after staining, to lyse red blood cells.

Flow cytometry was performed on an EPICS Coulter XL-MCL Flow Cytometer (BC). At least 20,000 events were acquired for extracellular and 100,000 events for intracellular staining. Data analysis was performed using the FlowJo V7.5 software (Tree Star Inc., Ashland, OR, USA).

### 4.5. Cytokines Measured in Culture Supernatants

Both inflammatory (IFN-γ, TNF-α, and IL-17A) and anti-inflammatory (IL-4 and IL-10) cytokines were measured in the supernatants of PBMC cultured with or without peptides. Measurement of cytokine levels was performed on a BD FACSArray Bioanalyzer using the cytometric bead array (CBA) assay (human Th1/Th2/Th17 Cytokine Kit, BD Biosciences). The ratio of anti-inflammatory/inflammatory cytokines was calculated in all cultures and the net change was determined to assess the shift towards a suppressor or effector phenotype after culture with each peptide.

### 4.6. Statistical Analysis

Comparisons between different groups of patients and controls were performed using the one-way analysis of variance (ANOVA) or the Kruskal-Wallis test, depending on the normality of the distribution of values. When the null hypothesis of the Kruskal-Wallis or ANOVA test was rejected, the Mann-Whitney test with Bonferroni correction was employed for the pairwise comparisons of the groups. The p values were calculated two-tailed and in all cases considered statistically significant if *p* was ≤0.05. Data were analyzed using the GraphPad Prism v.5.03 (San Diego, CA, USA).

## 5. Conclusions

In conclusion, in RRMS patients, different regulatory cell populations show a varied distribution according to the phase of the disease and treatment regimens. nTreg levels were lower in the patients in the acute phase of the disease before therapy or under methylprednisolone treatment, but were restored to HC-levels in patients in the acute phase receiving interferon. Patients in remission receiving no treatment had HC-levels of nTregs, whereas under natalizumab-treatment their nTreg levels were decreased to acute phase levels. Patients receiving interferon had the highest levels of CD3^+^CD4^+^HLA^−^G^+^ and CD8^+^ Tregs, whereas patients in the acute phase receiving methylprednisolone the lowest. Patients in remission had the highest levels of iNKT cells and patients in remission under natalizumab treatment the highest levels of CD56^bright^ cells. nTregs, but not the other regulatory cells studied, exhibited suppressive activity when cultured with at least one myelin peptide antigen, i.e., they retained their function when they recognized their cognate antigen.

## Figures and Tables

**Table 1:** Demographic and clinical characteristics of MS patients and controls.

Group	N (M/F)	Age (Years)	Disease Duration (Years)	EDSS
HC	45 (19/26)	36.31 ± 9.26	NA	NA
RRMS patients	83 (33/50)	37.53 ± 10.65	4.31 ± 3.80	2.41 ± 1.89
AP-noRx	13 (4/9)	35.54 ± 9.46	1.76 ± 2.66	1.61 ± 1.74
AP-MP	17 (8/9)	32.82 ± 10.32	1.13 ± 1.55	1.55 ± 1.23
AP-IFN	12 (4/8)	33.75 ± 9.03	3.62 ± 1.79	2.83 ± 1.55
Rem-noRx	15 (6/9)	42.47 ± 13.44	5.75 ± 3.86	2.36 ± 2.09
Rem-NATA	26 (11/15)	39.35 ± 8.71	6.93 ± 3.24	3.34 ± 1.82

Data are given as mean ± SD. HC, healthy controls; AP, patients in the acute phase of the disease; Rx, treatment; Rem, patients in remission; MP, methylprednisolone; IFN, interferon β; NATA, natalizumab; NA, not applicable; N, number of subjects; M, male; F, female; EDSS, expanded disability status scale.

**Table 2:** Peptides used for the functional assays.

Peptide	Description	Sequence
PEP1	Rat MOG_35–55_ epitope conjugated with oxidized mannan	H–Met^35^–Glu–Val–Gly–Trp–Tyr–Arg–Ser–Pro–Phe–Ser–Arg–Val–Val–His–Leu–Tyr–Arg–Asn–Gly–Lys^55^–OH
PEP2	Rat MOG_35–55_ epitope conjugated with reduced mannan	
PEP3	Human MOG_35–55_ epitope conjugated with oxidized mannan	H–Met^35^–Glu–Val–Gly–Trp–Tyr–Arg–Pro–Pro–Phe–Ser–Arg–Val–Val–His–Leu–Tyr–Arg–Asn–Gly–Lys^55^–OH
PEP4	Human MOG_35–55_ epitope conjugated with reduced mannan	
cP7	Citrullinated human MBP_87–99_ epitope: Cyclo(87–99) [Cit^91^, Ala^96^, Cit^97^] MBP_87–99_	Cyclo(87–99) Val–His–Phe–Phe–Cit^91^–Asn–Ile–Val–Thr–Ala^96^–Cit^97^–Thr–Pro
